# Connexin 43 expression is associated with increased malignancy in prostate cancer cell lines and functions to promote migration

**DOI:** 10.18632/oncotarget.3449

**Published:** 2015-03-23

**Authors:** Ao Zhang, Masahiro Hitomi, Noah Bar-Shain, Zafardjan Dalimov, Leigh Ellis, Kiran K. Velpula, Gail C. Fraizer, Robert G. Gourdie, Justin D. Lathia

**Affiliations:** ^1^ Cleveland Clinic Lerner College of Medicine at Case Western Reserve University, Cleveland, OH, 44195, USA; ^2^ Department of Cellular and Molecular Medicine, Lerner Research Institute, Cleveland Clinic, Cleveland, OH, 44195, USA; ^3^ Genitourinary Program, Department of Pharmacology & Therapeutics, Roswell Park Cancer Institute, Buffalo NY, 14263, USA; ^4^ Department of Cancer Biology and Pharmacology, University of Illinois College of Medicine at Peoria, Peoria, IL, 61656, USA; ^5^ Department of Biological Sciences, Kent State University, Kent, OH, 44242, USA; ^6^ Center for Heart and Regenerative Medicine, Virginia Tech Carilion Research Institute, Roanoke, VA, 24016, USA; ^7^ Case Comprehensive Cancer Center, Cleveland, OH, 44106, USA

**Keywords:** Cx43, prostate cancer, invasion, gap junction independent function

## Abstract

Impaired expression of connexins, the gap junction subunits that facilitate direct cell-cell communication, have been implicated in prostate cancer growth. To elucidate the crucial role of connexins in prostate cancer progression, we performed a systematic quantitative RT-PCR screening of connexin expression in four representative prostate cancer cell lines across the spectrum of malignancy. Transcripts of several connexin subunits were detected in all four cell lines, and connexin 43 (Cx43) showed marked elevation at both RNA and protein levels in cells with increased metastatic potential. Analysis of gap-junction-mediated intercellular communication revealed homocellular coupling in PC-3 cells, which had the highest Cx43 expression, with minimal coupling in LNCaP cells where Cx43 expression was very low. Treatment with the gap junction inhibitor carbenoxolone or connexin mimetic peptide ACT-1 did not impair cell growth, suggesting that growth is independent of functional gap junctions. PC-3 cells with Cx43 expression reduced by shRNA showed decreased migration in monolayer wound healing assay, as well as decreased transwell invasion capacities when compared to control cells expressing non-targeting shRNA. These results, together with the correlation between Cx43 expression levels and the metastatic capacity of the cell lines, suggest a role of Cx43 in prostate cancer invasion and metastasis.

## INTRODUCTION

The factors determining progression and metastasis of solid tumors remain elusive, but exchange of signals and coordinated intercellular communication between different types of cells within the heterogeneous cancer cell population and/or between the cancer cells and their microenvironment are believed to play an important role. Gap junctions, composed of proteins of the connexin family, are specialized transmembrane channels directly connecting cytoplasm of adjacent cells. Currently there are over 20 connexin isoforms that have been identified in mammals [1], and they share the same structural configuration of four transmembrane α-helical domains, three loops that connect them, and two intracellular tails. Six connexin subunits form a hemichannel called connexon, and two of which further assemble into a homotypic or heterotypic gap junction. Small (<1 kDa) molecules, such as glucose, ATP, and ions readily exchange between cells through gap-junction-mediated intercellular communication (GJIC). GJIC contributes to the maintenance of proper cellular communication and homeostasis, especially metabolic synchronicity, and their disruption is often associated with abnormal cell proliferation. Aberrant connexin expression is implicated in developmental abnormalities and multiple cancer types [2], but there has been increasing evidence for GJIC-independent roles of connexins on cell growth, differentiation and tumorigenicity [3]. These findings challenge the traditional view that connexins only function as structural components of gap junctions and suggest their potential roles as signaling molecules.

The relationship between connexins and cancer is complex. Connexins have traditionally been classified as tumor suppressor genes due to their down-regulation in various tumor types as well as their anti-proliferative effects when over expressed. However, this simplistic role has recently been challenged [4]. Instead of the simplified tumor-suppressive role, recent studies demonstrated that connexins are implicated in the multiple stages of cancer from initiation to progression and metastasis [4]. Although re-expression of connexins in both metastatic and non-metastatic cancer cell lines decreases tumorigenesis and favors the more benign mesenchymal to epithelial transitions [5, 6], connexins are also shown to facilitate tumor progression in late stage disease by promoting tumor cell extravasation and metastasis [7–9]. These studies highlight the stage-specific anti- or pro-tumorigenic functions of connexins during the development of cancer in various cellular and tissue contexts.

Prostate cancer (PC) is the most common noncutaneous cancer among men in the US and the second leading cause of cancer-related mortality [10]. Advanced prostate cancer commonly spreads to the bones, lungs and liver [11]. Several connexin isoforms, such as Cx26, Cx32 and Cx43 have been confirmed to be expressed in both normal prostate tissue and prostate carcinoma [12]. As in other solid tumors, connexins are also proposed to exhibit stage-specific functions in prostate cancer promotion and progression [13]. In the current study, we systematically surveyed connexin expression in four representative prostate cancer cell lines and found evidence for the correlation between increased Cx43 expression levels with prostate cancer cell line malignancy. We identified the existence of GJIC in PC-3 cells, but not in LNCaP cells, and demonstrated the growth of both cell types was GJIC independent, using pan-gap junction inhibitors. With shRNA mediated down-regulation of Cx43, we demonstrated the role of Cx43 expression in the migration and invasion of prostate cancer cells. Our data suggest that for prostate cancer cell lines, Cx43 expression increases the metastatic potential, but its expression is not required for cell proliferation.

## RESULTS

### Cx43 expression levels correlate with degrees of malignancy of prostate cancer cell lines

**Figure 1:**
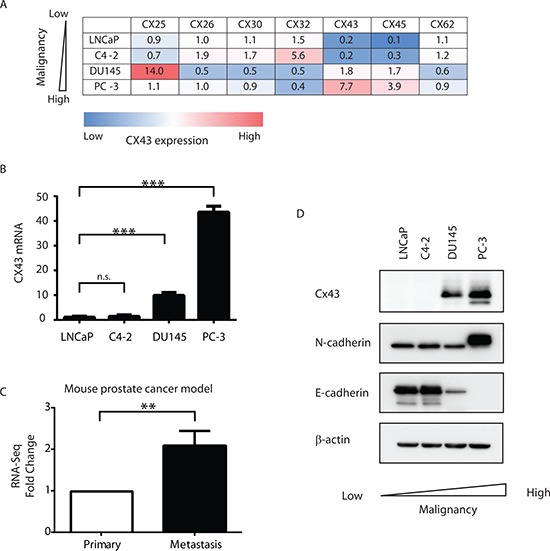
Cx43 expression is associated with increased malignancy in prostate cancer cells **(A)** Expression of connexin mRNA in prostate cancer cell lines (LNCaP, C4-2, DU145 and PC-3) measured by qRT-PCR. A summary of relative connexin mRNA levels in the indicated cells. Only connexin isoforms with Ct ≤ 35 are included. The color scheme shows qualitative information (blue: low expression; red: high expression). The insert numbers represent the relative levels of each connexin isoform with the median value in each column set to 1. Three replicas were analyzed for each cell line and the average values were used for the table. All the errors were <10% and are not shown for simplicity. GAPDH mRNA expression levels were used as reference in data analysis with the 2-^ΔΔ^CT method. **(B)** Quantification of *CX43* mRNA expression, using the 2^−ΔΔCT^ method with GAPDH mRNA as reference values. The quantity of *CX43* mRNA in LNCaP cells was set as 1. n.s., not significant. *P* values were calculated using one-way ANOVA and Dunnett's post-test. ***P ≤ 0.001. **(C)** Fold change of *CX43* expression from RNA-seq analysis of a mouse prostate cancer model. *CX43* expression was up-regulated in metastatic prostate cancer compared to primary cancer in the same mouse. The expression of *CX43* in primary tumors was set as reference. **P ≤ 0.01. **(D)** Cx43, E-Cadherin and N-Cadherin protein levels in prostate cancer cell lines detected by Western blot using their respective antibodies. β-actin serves as loading control.

It has been reported previously that various connexin subunits exhibited different expression levels based on stages of prostate cancer progression from normal tissues to primary cancer and to metastasis [4, 13]. Using qRT-PCR, we first systematically analyzed connexin expression in four prostate cancer cells lines with varied malignancy. LNCaP (together with its derivative C4-2), DU145 and PC-3 represent prostate cancer cells with low, intermediate and high metastatic potential [14]. Examination of all the human connexin isoforms revealed that *CX43* expression was consistently associated with the degree of malignancy (Figure 1A). Both LNCaP and C4-2 cells with low grade metastatic potentials showed low level of *CX43* expression compared to intermediately to highly metastatic cell lines DU145 and PC-3 cells that presented with about 10- and 40-fold increase of *CX43* mRNA levels, respectively (Figure 1B). We next investigated *CX43* expression in a mouse model of prostate cancer with spontaneous metastasis generated by orthotopic implantation of Myc-CaP cells [15]. We detected more than 2-fold increase of *CX43* in metastatic cancer in lymphatic tissues compared with primary cancer in the same mouse with RNA-seq (Figure 1C). To verify the Cx43 expression differences between cell lines at the protein level, we performed Western blot using a polyclonal antibody against Cx43 protein and detected endogenous Cx43 protein expression was also elevated in DU145 and PC-3 cells compared with LNCaP and C4-2 cells as expected (Figure 1D). It has been shown previously that Cx43 formed a complex with cadherin proteins and we examined the protein expression of both N-cadherin and E-cadherin, two proteins implicated in the epithelial to mesenchymal transition (EMT) process in cancer progression [16]. We found Cx43 expression levels were associated with increased expression of N-Cadherin and decreased expression of E-Cadherin. These data suggest that Cx43 is associated with increased malignancy and progression in prostate cancer cells.

### Pharmacological manipulation of gap junction activity does not affect cell growth

**Figure 2:**
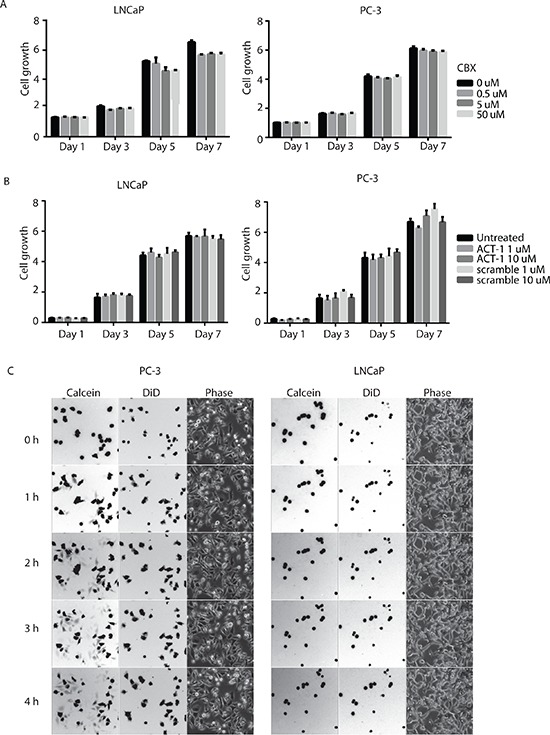
Gap-junction-mediated intercellular communication is not required for growth of prostate cancer cells **(A)** Growth of neither LNCaP nor PC-3 cells was affected by pharmacological inhibition of gap-junctions with increasing concentration of CBX. Cell growth was measured by CellTiter-Glo® Luminescent Cell Viability Assay and the units are arbitrary. The experiments were performed in triplicate and representative results are shown. No statistically significance difference was detected. **(B)** Growth of neither LNCaP nor PC-3 cells was affected by the connexin mimetic peptide ACT-1. The experiments were performed similarly and analyzed as in (A). No statistically significance difference was detected. **(C)** Gap-junctions that mediate intracellular transfer of calcein AM dye are functional in PC-3 (left panel), but not LNCaP (right panel) cells. Fluorescent (calcein), far-red (DiD) and phase images were captured at the same time (0-4 hours) with a Leica DMI6000B microscope with a 10x objective lens.

The most well established function of connexin proteins is to form gap junctions between adjacent cells and mediate direct intercellular communication. To assess whether gap junction activity is necessary for prostate cancer cell growth, we first utilized a pharmacological pan-gap junction inhibitor carbenoxolone (CBX). CBX is a derivative of 18-glycyrrhetinic acid that blocks connexin hemichannels and gap junctions [17]. Both LNCaP and PC-3 cells were treated with increasing amounts of CBX (0.5 μM, 5 μM and 50 μM), and no difference in cell growth was detected between CBX treated cells and control cells (Figure 2A). To evaluate if increasing CX43 channel accretion into gap junction plaque affects prostate cancer cell growth, we utilized the ACT-1 (alpha connexin 43 carboxy-terminus peptide 1), a 25 mer synthetic cell membrane permeable peptide derived from Cx43 that specifically targets the interaction between Cx43 and zonula occuludens (ZO)-1 and releases the inhibition of ZO-1 on the structural organization of Cx43 gap junctions [18]. Both LNCaP and PC-3 cells were treated with ACT-1 peptide or a scramble control peptide, and their growth was monitored for a week. No statistically significant difference was observed (Figure 2B). These data demonstrate that growth of prostate cancer cells LNCaP and PC-3 is not affected by gap junction inhibition or increase in Cx43 gap junction extent by ACT-1 peptide.

### PC-3 cells maintain functional gap junction dependent intracellular communication

To rule out the possibility that lack of response to gap junction inhibition and modulation is due to lack of functional GJIC, we determined gap junction activity in these cells. Specifically, we used a dye transfer assay and followed the movement of a diffusible fluorescent dye, calcein, between dye-loaded donor cells and recipient cells of the same type. As monitored by time-lapse microscopy, the calcein AM dye diffused into recipient cells starting at 1 hour after contact with donor cells in PC-3 cell cultures and the transfer continued for the entire observation period (4 hours) (Figure 2C, left panel). The DiD dye remained in the donor cells because it is not transferrable to adjacent cells once incorporated into cell membrane. In sharp contrast, limited dye transfer was detected in LNCaP cells throughout the same observation period (Figure 2C, right panel). These data suggest that functional gap junctions are present in PC-3 cells and are limited in LNCaP cells.

### Cx43 is involved in migration and invasion of PC-3 cells, but not cell growth

**Figure 3:**
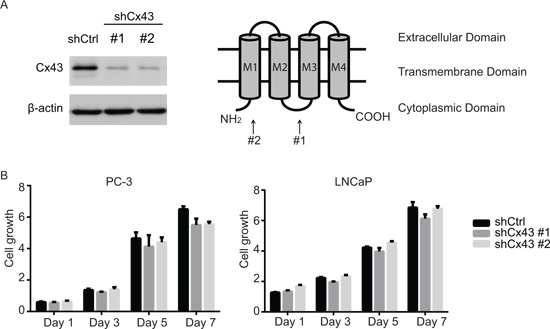
Suppression of Cx43 does not affect growth of prostate cancer cells **(A)** Left panel, down-regulation of Cx43 in PC-3 cells by two different shRNA constructs confirmed with Western blot. Right panel, topological structure of Cx43 protein. The locations that the two shRNA constructs target are indicated. **(B)** Cell growth was measured as described for Figure 2. Growth of both PC-3 and LNCaP cells with shRNA knocked-down Cx43 was similar compared to that of control cells. The experiments were performed in triplicate and representative results are shown. No statistically significance difference was detected.

Despite the lack of response to gap junction inhibitors, the correlation between the levels of Cx43 expression and the spectrum of malignancy of prostate cancer cell lines strongly suggests a critical role of Cx43 in prostate cancer progression. We next, therefore, assessed functions of Cx43 by using lentivirus delivered shRNA to stably down-regulate Cx43 expression in PC-3 cells that exhibited the highest endogenous level of Cx43 of all four prostate cancer cells lines investigated in the current study. We used two different shRNA constructs that targeted two different regions of Cx43: one for the sequence encoding the first cytoplasmic loop between the second and third transmembrane domains, and the other for the region encoding the cytoplasmic tail at the N terminus. Both constructs successfully decreased Cx43 expression in PC-3 cells compared with non-targeting, scramble shRNA control based on protein blot (Figure 3A). Using the same cell growth assay as described earlier, we did not observe any significant change in cell growth of PC-3 cells by any of Cx43 targeting shRNA constructs, except for day 7 when both constructs showed a minor (10%) decrease in cell growth (Figure 3B, left panel). To rule out any potential off-target effects of the two Cx43 shRNA constructs, we also generated LNCaP cells that stably expressed each of targeting shRNA or the non-targeting shRNA. Based on our observation that LNCaP cells have less than detectable Cx43 protein levels (Figure 1C), we expected shRNA Cx43 targeting would have no significant effect. Indeed, no difference in cell growth was observed between the three resulting LNCaP cell lines, suggesting the lack of off-target effects of our Cx43 shRNA reduction approach (Figure 3B, right panel). Taken together, these data suggest that Cx43 is not necessary for growth of prostate cancer cell lines, PC-3 and LNCaP.

**Figure 4:**
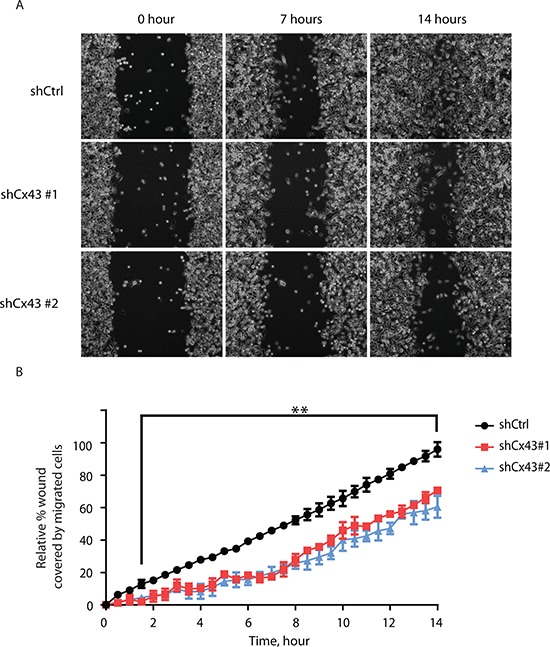
Suppression of Cx43 inhibits migration of PC-3 cells **(A)** Representative phase microscopic images of control PC-3 cells and PC-3 cells with down-regulated Cx43 at various times after monolayer wounding (0, 7, and 14 hours). **(B)** Quantification of cell migration using the monolayer wound healing assay. Percentage of wound covered by migrated cells was determined by comparing the area occupied by migrated Cx43 knocked-down PC-3 cells to that of control cells at the end of the observation period (14 hours), which was used as reference. The areas covered by migrated cells from three independent microscopic fields were quantified by Image-J. Relative percentage of wound covered by migrated control cells and two Cx43 knock-down lines at each time point was statistically significant at the 0.01 level starting from 90 minutes to the end of the experiment based on two-way ANOVA and Tukey's multiple comparisons test. **P ≤ 0.01.

**Figure 5:**
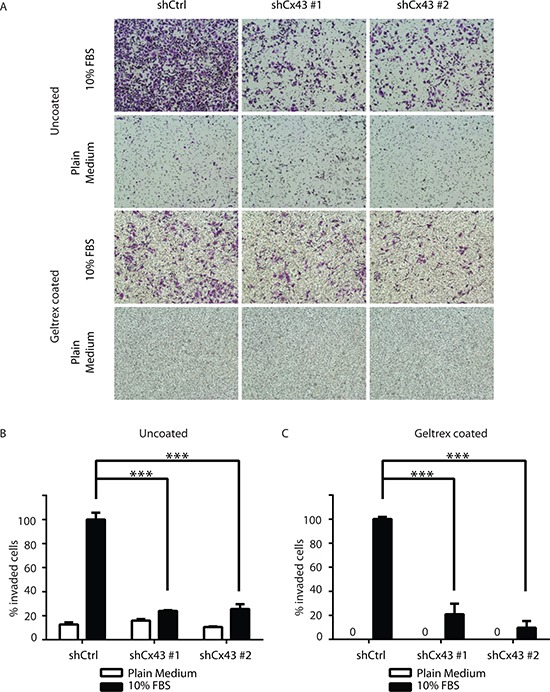
Cx43 is required for transwell invasion potential of PC-3 cells **(A)** Transwell invasion across uncoated (upper panel) and Geltrex coated (lower panel) inserts of control PC-3 cells and PC-3 cells with down-regulated Cx43 in the presence or absence of FBS, a chemoattractant. Cells invaded through the transwell membrane barrier were fixed and stained with crystal violet. Images were captured with a Leica DMI4000B microscope with a 10x objective lens. A representative microscopic field of each condition is shown. **(B)** Quantitation of the percentage of invaded cells across uncoated membrane. The invaded cells were quantified by determining the area of crystal violet staining using Image-J. The average area size from three independent microscopic fields was presented. Invasion of control shRNA transduced PC-3 cells in the presence of 10% FBS containing medium was used as reference. *P* values were calculated using one-way ANOVA and Dunnett's post-test. ***P ≤ 0.001. **(C)** Quantification of the percentage of invaded cells across Geltrex coated membrane. Data were analyzed and plotted similarly as described in **(B)**. ***P ≤ 0.001.

In addition to proliferative capacity, metastatic potential also contributes to cancer malignancy. Therefore we next evaluated whether Cx43 was involved in the migration and invasion of PC-3 cells. Using the monolayer wound healing assay with time-lapse microscopy, we found both lines of PC-3 cells with down-regulated Cx43 exhibited reduced migration capacity compared to PC-3 cells transduced with non-targeting shRNA in a time-dependent manner (Figure 4A). At the end of 14 hours, both lines of Cx43 down-regulated cells covered only about 50% to 60% of surface area covered by control PC-3 cells (Figure 4B). The difference in migratory abilities was further confirmed in the transwell invasion assay with 10% FBS containing medium as the attractant (Figure 5A). Unlike the monolayer wound healing assay that is useful to determine the migratory ability of whole cell masses, the transwell assay provides information about the invasiveness of the migrating single cells that move directionally in response to chemoattractants, such as growth factors, across membrane through pores with diameter (8 μM) well below that of the cells in suspension [19]. Both lines of PC-3 cells with down-regulated Cx43 exhibited dramatically reduced migration capacity compared to PC-3 cells transduced with non-targeting shRNA (Figure 5A, upper panel). When migrated cells were stained, the stained area, which was approximately proportional to number of stained cells, was quantified using Image-J. We detected about an 80% reduction of the number of migrated cells when Cx43 was down-regulated (Figure 5B). The invasion of basement membranes by cancer cells represents an important step of a group of coordinated cellular processes that eventually lead to successful establishment of tumor metastasis [20]. We therefore investigated the invasion of PC-3 cells across transwell insert membranes coated with a layer of extracellular matrix, Geltrex, in a similar transwell assay, which mimicked the process of extracellular matrix invasion of tumor cells. No invasion occurred in the absence of 10% FBS as attractant (Figure 5A. lower panel). Further quantification showed statistically significantly decreased invasion of PC-3 cells with down-regulated Cx43 compared to the control cells when 10% FBS was present (Figure 5C). These data suggest that Cx43 potentially contributes to metastasis of prostate cancer cells by enhancing cell migration and invasion.

## DISCUSSION

The role of connexins during prostate cancer development and progression is still not well understood [13]. Impaired expression and function of connexins has been detected in prostate cancer cells and tissues from patients, while restoration of connexin expression is crucial requirement for invasive phenotype. All these observations support the notion that connexin dysfunction has a complex and stage-specific effect during the natural course of prostate cancer progression. Our data suggest that Cx43 expression correlates with malignant phenotype and its expression is associated with EMT. While we could not detect gap junction activity in LNCaP cells, there does seem to be functional gap junction in PC-3 cells. However, pharmacological manipulation of this communication pathway does not seem to affect cell growth and survival. On the other hand, our data suggest that Cx43 is involved in migration. These results strongly imply that in prostate cancer cells, connexin proteins are not required for cell growth, but are capable of mediating other pathophysiological processes, such as cell migration.

Of all known connexin isoforms, Cx43 is the most well-studied isoform playing a critical role in cardiovascular development and electrical coupling between cardiomyocytes through homotypic gap-junction channels. It is widely expressed in various tissues and is the most predominant connexin isoform expressed in most tissue culture cell lines [21]. In prostate, Cx43 is found predominantly in both undifferentiated and mature basal cells of the prostate epithelium and not in the luminal cells [22, 23]. We found higher expression levels of Cx43 in androgen receptor negative prostate cancer cell lines PC-3 and DU145 compared with their androgen receptor positive counterparts LNCaP and C4-2 [24]. Interestingly, castration induces Cx43 mRNA and protein expression in rat ventral prostate tissue, and this effect is abolished by administration of dihydrotestosterone [25]. This result suggests an association, possibly an inverse relationship, between androgen receptor signaling and Cx43 expression in prostate cancer cells.

The primary function of connexins is to form conduits between neighboring cells that mediate the intercellular exchange of small cytoplasmic molecules. Our data indicates that this communication machinery can function in some prostate cancer cells. However, inhibition of GJIC by the pharmacological reagent carbenoxolone did not exert detectable effect to inhibit cell proliferation. Furthermore, increasing Cx43 hemichannel aggregation into gap junction plaques with ACT-1 did not increase cell growth either. These results suggest GJIC might be dispensable for prostate cancer proliferation. Indeed we did not detect any intercellular dye transfer in another prostate cancer cell line LNCaP that lacks Cx43, suggesting prostate cancer cells might use alternative mechanisms other than GJIC to coordinate and maintain a complex and stable system required for disease progression.

We observed a positive correlation between Cx43 and N-cadherin expression in prostate cancer cells. Cx43 and N-cadherin were shown to colocalize within a multiprotein complex that is required for gap junction formation [16]. This complex collectively modulates cell motility through downstream p120 catenin signaling [26]. The interaction between Cx43 and N-cadherin could explain the decreased migration of PC-3 cells when Cx43 was down-regulated by shRNA. However, this might be just one of many possible mechanisms explaining the role of Cx43 in cell motility. Cx43 is one of the target genes that was identified as involved in cell migration in an RNA interference knockdown screen [27]. It is capable of recruiting multiple adaptor proteins that link to the cytoskeleton [28], and its expression influences the adhesiveness of cells and the directionality of multiple cellular processes, so that cells can move in an organized manner by rearranging their cytoskeleton [29]. It is noteworthy that the effect of connexins, including Cx43, on migration involves integrated function of several domains of connexin proteins, and the overall effect could be independent of their channel functions [4]. It has been shown that during brain development, connexin proteins mediate the migration of radial glia to the cerebral cortex using a GJIC independent mechanism [30]. The finding that Cx43 plays an important role in the migration and invasion of prostate cancer is also consistent of previous findings that LNCaP cells overexpressing Cx43 exhibit increased bone metastasis, as well as, Cx43 expression is elevated in DU145 cells with increased invasiveness [31, 32].

Our results illustrated the involvement of Cx43 in migration and invasion of prostate cancer cells *in vitro*, and it is important to further explore the function of Cx43 *in vivo*. RNA-seq data of paired primary and metastatic tumor samples from the same mouse model revealed elevated *CX43* expression in lymphatic tissue metastasis compared to primary tumor, which suggests Cx43 plays a similar role in facilitating tumor migration and invasion *in vivo* as well. The expression of Cx43 protein also seems to be elevated in bone metastasis compared with primary adenocarcinoma from our pilot experiment with human prostate cancer tissue microarray (data not shown). These findings suggest increased expression of Cx43 correlates with prostate cancer metastasis, but given their preliminary nature, additional larger and systemic clinical studies are required.

In conclusion, we identified several connexin isoforms expressed in prostate cancer cells. Although connexin mediated GJIC was not required for the proliferation of prostate cancer cell lines, one connexin isoform, Cx43, was important for the migration and invasion of PC-3 cells. The exact mechanism of how Cx43 facilitates cell migration in prostate cancer cells requires further investigation, and understanding this pathway could make Cx43 a potential pharmacological target to prevent metastasis of prostate cancer.

## METHODS

### Cell culture and shRNA stable line generation

Prostate cancer cell lines LNCaP, C4-2, DU145, PC-3 were maintained in RPMI medium supplemented with 10% fetal bovine serum (FBS), 50 U/ml of penicillin, 50 μg/ml of streptomycin and 2 mM L-glutamine (Life Technologies). LNCaP was purchased from ATCC, C4-2 was kindly provided by Dr. Alex Almasan (Cleveland Clinic), and DU145 and PC-3 were kindly provided by Dr. Nima Sharifi (Cleveland Clinic). LNCaP and PC-3 cells with down-regulated Cx43 were prepared by transduction with lentivirus expressing Cx43 specific shRNA (Sigma, TRCN0000059773 and TRCN0000059775). Lentivirus was produced in 293T cells using standardized protocol with packaging plasmids as described previously [33], and viral particles containing conditioned medium was filtered through 0.45 μM PVDF membrane and directly used to infect prostate cancer cell lines in the presence of 8 μg/ml polybrene (Sigma). Twenty four hours after infection, cells were selected with puromycin (Sigma) at a final concentration of 3 μg/mL for PC-3 cells and 1 μg/mL for LNCaP cells. Surviving cells were pooled together and maintained in medium containing 1 μg/mL puromycin for both cell lines. Control cells were obtained in a similar manner with lentivirus expressing non-targeting shRNA (Sigma, SHC002).

### Cell growth assay

Cell growth assay was performed with CellTiter-Glo® Luminescent Cell Viability Assay (Promega) according to manufacturer's protocol. In brief, 5,000 cells were seeded into each well of 96-well opaque-walled plates in 100 μL RPMI containing carbenoxolone, ACT-1 peptide or control peptide. For each treatment condition, three replicate wells were prepared. Five plates were prepared, each for one time point. Immediately after cell plating (day 0), 100 μL of CellTiter-Glo® Reagent was added to each well of one plate and the contents were mixed on an orbital shaker for 10 minutes. Luminescence were recorded on a VICTOR3 Multilabel Plate Reader with an integration time of 1 second per well. The remaining four plates were incubated at 37°C with 5% CO_2_, with cell growth assay performed at day 1, day 3, day 5 and day 7. The luminescence results from day 0 served as the reference.

### Gap junction dye transport assay

To quantify homocellular gap junction-mediated intercellular transport, we used time-lapse video microscopy. Prostate cancer cell lines LNCaP and PC-3 were plated on 12-well plates the day before the assay and allowed to form a nearly confluent recipient monolayer. On the day of the assay, single-cell suspensions of LNCaP and PC-3 cells were prepared and loaded with calcein AM and DiD (Life Technologies) as donor cells, according to the manufacturer's instructions. After the free dyes were washed away, labelled donor cells were added onto their respective recipient cells, and time-lapse video microscopy was used to capture phase contrast, green fluorescent (for calcein), and far-red fluorescent (for DiD) images. Images were captured using a Leica DMI6000B microscope with a 10x objective lens. The signals observed outside of the DiD labeled donor cells indicated the dye was transported to neighboring recipient cells.

### Quantitative RT-PCR and immunoblot analysis

To determine the expression levels of connexins, primers for all known connexin types were synthesized (Supplementary table 1) for quantitative RT-PCR (qRT-PCR). Total RNA from prostate cancer cells was prepared with Trizol (Life Technologies) according to manufacturer's protocol. After contaminating DNA was removed using a Turbo DNA-free kit (Ambion), cDNA was synthesized using the High Capacity cDNA Reverse Transcription (RT) kit (Life Technologies). The resultant cDNA was amplified using Sybr Green PCR master mix (Life Technologies) on a StepOnePlus system according to manufacturer's protocol. Data were analyzed with the 2^−ΔΔCT^ method using the expression of GAPDH gene as reference if amplification of the target was detected below a background threshold (Ct ≤ 35). Details of the mouse prostate cancer spontaneous metastasis model and RNA-seq analysis were described elsewhere [15, 34].

For immunoblot analysis, cells were lysed in 10 mM Tris HCl, pH 7.4; 0.5% IGEPAL CA-630 (weight/volume); 150 mM NaCl; 1 mM EDTA; 2 mM sodium orthovanadate; 1 mM PMSF; and a 1:100 dilution of protease inhibitor cocktail for mammalian cells (Sigma). Total protein concentration of the lysate was estimated using Protein Assay Dye (Bio-Rad) with a BSA standard (Thermo) as reference. After denaturation according to Laemmli [35], 20 μg of total protein per sample was separated by electrophoresis on 10% SDS-polyacrylamide gel (SDS-PAGE). Separated proteins were electro-transferred to a PVDF membrane, and the molecule of interest was detected by immunoblotting using specific antibodies against Cx43 (Cell Signaling, 1:1000 dilution), E-cadherin (Millipore, 1:5000 dilution), N-cadherin (Millipore, 1:5000 dilution), and β-actin (Sigma, 1:5000 dilution). Blots were visualized by incubating with species-specific horseradish peroxidase (HRP)-conjugated secondary antibodies and ECL-plus (Thermo Scientific) substrate followed by capturing of the chemiluminescence signal with X-ray film.

### Wound healing assay

PC-3 cells were plated in a 6-well plate and allowed to reach 100% confluence overnight. A vertical wound was made with a 200 μl pipette tip through the cell monolayer in a sterile environment. Media and detached cell debris were aspirated and 2 ml fresh media was added to each well. The cultured cells were return to culture at 37°C with 5% CO_2_ for 14 hours. Serials of images were captured every 30 minutes using a Leica DMI6000B microscope with a 10x objective lens. The surface areas of the wound and areas subsequently covered by migrating cells were quantified with Image J software. Percentage of wound covered by migrated cells was determined by comparing the area occupied by each cell line at indicted time points to that of control cells at the end of the observation period (14 hours), which was set as reference of 100% coverage.

### Transwell invasion assay with or without extracellular matrix barrier

For the transwell migration assay, PC-3 cells cultured in serum free RPMI for overnight were harvested and suspended in serum free RPMI at a final concentration of 5×10^5^/ml. In a 24-Multiwell insert system with 8 micron pores (BD Falcon), 300 μl of cell suspension was added to each insert well and 1400 μl of RPMI with or without 10% FBS as chemoattractant was added to each lower well. After 24-hour incubation, migratory cells were fixed with 3.7% paraformaldehyde for 5 minutes and stained with 1% crystal violet solution. The invasion assay was performed similarly except each insert was precoated with a thin layer of Geltrex LDEV-free reduced growth factor basement membrane matrix (Life Technologies) diluted with serum free medium to a final concentration of 6 mg/ml. Cells were allowed to incubate for 48 hours before fixation and staining. Microscopy pictures were taken for each well at three randomly picked fields using a Leica DMI4000B microscope with a 10x objective lens and the stained area, which is approximately proportional to the number of stained cells, for each field was quantified with Image J software. Percentage of invading cells was determined by comparing the stained area of migrated Cx43 knocked-down cells to that of control cells, which was used as reference.

### Statistical analysis

Values reported in the results are mean values +/− standard deviation. Unless otherwise stated, one-way ANOVA was used to calculate statistical significance with Dunnett's post-test; *p* values are detailed in the text and figure legends. Two-way ANOVA and Tukey's multiple comparisons test was used to compare the relative percentage of wound covered by migrated control cells and two Cx43 knock-down lines at each time point in the monolayer wound healing assay. *P* values less than 0.05 are considered as statistically significant.

## SUPPLEMENTARY TABLE


