# Correction: Connexin 43 expression is associated with increased malignancy in prostate cancer cell lines and functions to promote migration

**DOI:** 10.18632/oncotarget.28411

**Published:** 2023-05-04

**Authors:** Ao Zhang, Masahiro Hitomi, Noah Bar-Shain, Zafardjan Dalimov, Leigh Ellis, Kiran K. Velpula, Gail C. Fraizer, Robert G. Gourdie, Justin D. Lathia

**Affiliations:** ^1^Cleveland Clinic Lerner College of Medicine at Case Western Reserve University, Cleveland, OH, 44195, USA; ^2^Department of Cellular and Molecular Medicine, Lerner Research Institute, Cleveland Clinic, Cleveland, OH, 44195, USA; ^3^Genitourinary Program, Department of Pharmacology and Therapeutics, Roswell Park Cancer Institute, Buffalo NY, 14263, USA; ^4^Department of Cancer Biology and Pharmacology, University of Illinois College of Medicine at Peoria, Peoria, IL, 61656, USA; ^5^Department of Biological Sciences, Kent State University, Kent, OH, 44242, USA; ^6^Center for Heart and Regenerative Medicine, Virginia Tech Carilion Research Institute, Roanoke, VA, 24016, USA; ^7^Case Comprehensive Cancer Center, Cleveland, OH, 44106, USA

**This article has been corrected:** Due to errors during figure assembly, the control images used in the bottom row of Figure 5A contain a partial, accidental duplication. The corrected Figure 5, obtained using the original data, is shown below. The authors declare that these corrections do not change the results or conclusions of this paper.

Original article: Oncotarget. 2015; 6:11640–11651. 11640-11651. https://doi.org/10.18632/oncotarget.3449

**Figure 5:**
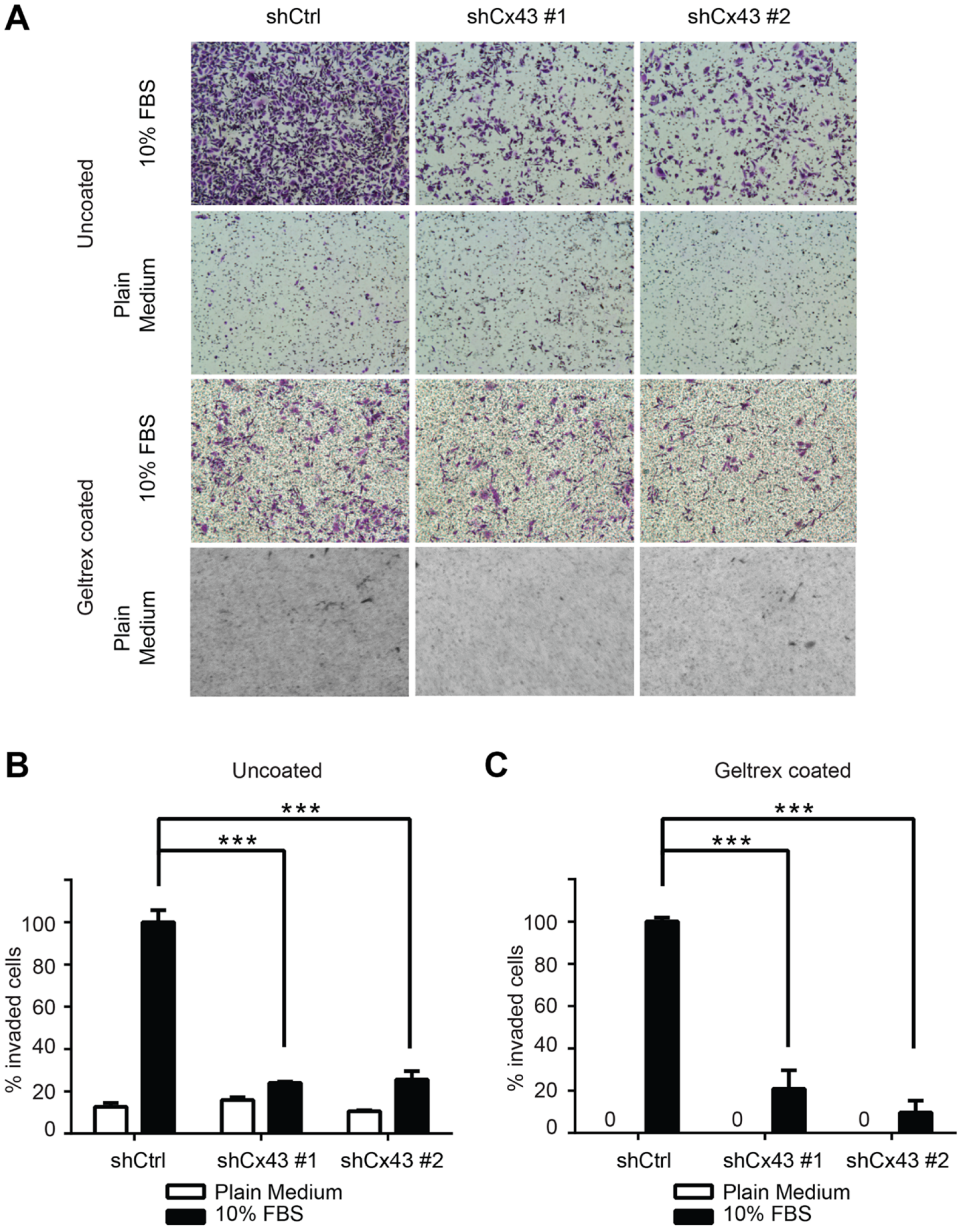
Cx43 is required for transwell invasion potential of PC-3 cells. (**A**) Transwell invasion across uncoated (upper panel) and Geltrex coated (lower panel) inserts of control PC-3 cells and PC-3 cells with down-regulated Cx43 in the presence or absence of FBS, a chemoattractant. Cells invaded through the transwell membrane barrier were fixed and stained with crystal violet. Images were captured with a Leica DMI4000B microscope with a 10x objective lens. A representative microscopic field of each condition is shown. (**B**) Quantitation of the percentage of invaded cells across uncoated membrane. The invaded cells were quantified by determining the area of crystal violet staining using Image-J. The average area size from three independent microscopic fields was presented. Invasion of control shRNA transduced PC-3 cells in the presence of 10% FBS containing medium was used as reference. P values were calculated using one-way ANOVA and Dunnett’s post-test. ^***^ *P* ≤ 0.001. (**C**) Quantification of the percentage of invaded cells across Geltrex coated membrane. Data were analyzed and plotted similarly as described in (B). ^***^ *P* ≤ 0.001.

