# Supplementary material for: Connexin 43 expression is associated with increased malignancy in prostate cancer cell lines and functions to promote migration
Source: Oncotarget. 2015 Mar 23;6(13):11640–51. doi: 10.18632/oncotarget.3449 (PMC4484482; doi:10.18632/oncotarget.3449)
Supplement: Supplementary file 1 [file oncotarget-06-11640-s001.pdf]

SUPPLEMENTARY TABLE

Supplementary Table S1: Sequence of qRT-PCR primers for connexin isoforms

**Cx25**	**NM_198568.2**
Forward	5′- ATG AGT CTG GTG AGC CAA GA -3′
Reverse	5′-CAC TCA GGA GAT CTC TGA GG -3′
**Cx26**	NM_004004.5
Forward	5′-TTA AAA GGC GCC ACG GCG GGA-3′
Reverse	5′-ATC CAT CTT CTA CTC TGG GC-3′
**Cx30**	NM_001110219.2
Forward	5′- TCT GAA ACA CTG GCA GTC GT -3′
Reverse	5′-AGA TGA CTG TGA TCC TCT CC -3′
**Cx30.2**	NM_181538.2
Forward	5′- CAC AAG AAA GCA ACC GAT AG -3′
Reverse	5′-AGT TCC CCA AAG TTC ATC TC -3′
**Cx30.3**	NM_153212.2
Forward	5′- AAG GAA GCA GCT ACT GGA CC -3′
Reverse	5′-CCC CTG TCC TCA TTT CAC AA -3′
**Cx31**	NM_024009.2
Forward	5′- TCT GTG TGC ACC ACT GCT GA -3′
Reverse	5′- AGT GTC TTC CAG TCC ATG GC-3′
**Cx31.1**	NM_005268.2
Forward	5′- AGC TGC TTG CTG AGT CCT AT -3′
Reverse	5′-AGT ACT TGT TGA CCC CAC TC -3′
**Cx31.9**	NM_152219.3
Forward	5′- TCT CAG GAG ATG TGG AGG CT -3′
Reverse	5′-GCA ACT CCG CTT TCA TAA CT -3′
**Cx32**	NM_001097642.2
Forward	5′- ACA TTC TCT GGG AAA GGG CA -3′
Reverse	5′-TGA AGA TGA AGA TGA CCG AG -3′
**Cx36**	NM_020660.1
Forward	5′-TGC AGC AGC ACT CCA CTA TGA T-3′
Reverse	5′-CAG GGT GTT GCA CAC AAA CA-3′
**Cx37**	NM_002060.2
Forward	5′-TCA CTC CGG CCA TCG T -3′
Reverse	5′-CAC CGT CAG CCA GAT CTT AC-3′
**Cx40**	NM_005266.5
Forward	5′- GGA ACA ACT GAC AGG CTC AA -3′
Reverse	5′-CCA GGG AAC AGA TGC CAA AA-3′

(Continued)

**Cx25**	**NM_198568.2**
**Cx40.1**	NM_153368.2
Forward	5′- GAC TTG CTA GGG TTT CTC AT -3′
Reverse	5′-TGT TGC AGA CAA ACC TCT CC -3′
**Cx43**	NM_000165.3
Forward	5′- ACT TGG CGT GAC TTC ACT AC -3′
Reverse	5′-TGA AAA GTA CTG ACA GCC AC-3′
**Cx45**	NM_005497.3
Forward	5′- TGG GTA ACC GAA GTT CTG GA -3′
Reverse	5′-TGT AAG GAC GAT CCG GAA GA -3′
**Cx46**	NM_021954.3
Forward	5′- CGC ACG TGT GAA AGG AAT TC-3′
Reverse	5′- GTC TTC CCA GAA AGC TCC AG-3′
**Cx47**	NM_020435.3
Forward	5′- TTG TGC TTG GTG GTG AGA GG-3′
Reverse	5′-GGA AGC TCC AGC TCA TGT TG -3′
**Cx50**	NM_005267.4
Forward	5′- ACA TAT TTC TCC GTG GGA CA -3′
Reverse	5′-GGA TCC GGA AGA TGA AAA GC -3′
**Cx59**	NM_030772.4
Forward	5′- CCA GCA AAG GGA CAA ATT CA -3′
Reverse	5′- TTC CAT TCT GAA GGG AGC AC-3′
**Cx62**	NM_032602.1
Forward	5′- GGA ACT TAT TGG GTG GCA TC -3′
Reverse	5′-TCA TCC CAG ACA TCC TCA GC -3′
**GAPDH**	NM_001256799
Forward	5′- GAA GGT GAA GGT CGG AGT C- 3′
Reverse	5′- GAA GAT GGT GAT GGG ATT TC -3′
